# Topical latanoprost causes posterior movement of lens in a patient with exfoliation syndrome and subluxated lens: a case report

**DOI:** 10.1186/1752-1947-1-172

**Published:** 2007-12-05

**Authors:** Takashi Kanamoto, Michiya Takamatsu, Yoshiaki Kiuchi

**Affiliations:** 1 Department of Ophthalmology and Visual Sciences, Graduate School of Biomedical Sciences, Hiroshima University, Japan

## Abstract

**Introduction:**

To report the effect of topical latanoprost on the position of a subluxated lens.

**Case presentation:**

After 0.005% latanoprost was administered topically to a patient with ocular hypertension due to a pseudoexfoliation syndrome and a subluxated lens, the position of the lens was examined by slit-lamp biomicroscopy, and the ciliary body thickness by ultrasound biomicroscopy. The lens had moved posteriorly, and the thickness of the ciliary body had decreased after the latanoprost.

**Conclusion:**

We suggest that the decrease in the thickness of the ciliary body resulted in an increase in the tension of the zonule of Zinn fibers, thus pulling the subluxated lens posteriorly.

## Case presentation

An 80-year-old woman complained of visual disturbances in her right eye that began in July 2002. She did not have a history of any systemic illness, and there was no family medical history of any disease. In 2001, she had undergone a peripheral iridotomy on the right eye for angle closure glaucoma, and she developed the pseudoexfoliation syndrome. Her postoperative intraocular pressure (IOP) in the right eye was 12 mmHg. The depth of the anterior chamber of the left eye was normal and the IOP was 11 mmHg.

In May 2002, although the IOP in her left eye was 12 mmHg, the right IOP was 22 mmHg, and we concluded that she had ocular hypertension secondary to the pseudoexfoliation syndrome. We began topical latanoprost in the right eye, and the IOP decreased to 13 mmHg by June. The IOP in the left eye remained at 12 mmHg. In July, she returned reporting visual disturbances and monocular double vision. The lens was partially dislocated in the right eye. At this time, the IOP in the right eye was 20 mmHg and the left eye was 10 mmHg. The right lens had a mild cataract. There were no clear glaucomatous changes in the optic discs, and no other specific findings. The Goldman perimetric fields were full.

**Figure 1:**
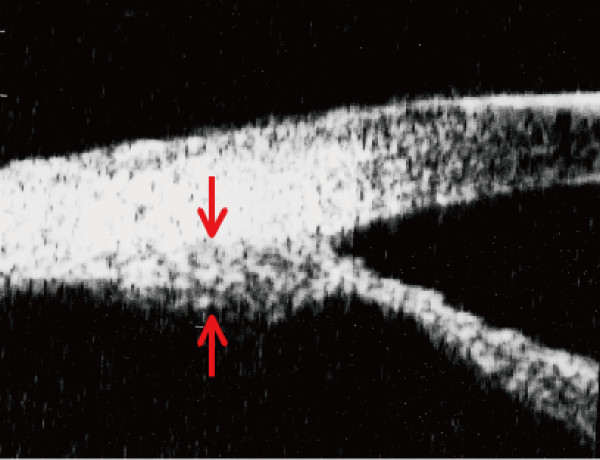
Measurement of the thickness of ciliary body by UBM (arrows).

To examine the effect of latanoprost on the position of the lens, we stopped the latanoprost for two weeks. The IOP was measured with a Goldman applanation tonometer one hour before and after topical latanoprost, and the position of the lens was assessed by slit-lamp biomicroscopy. In addition, ultrasound biomicroscopy (UBM) was performed to measure any changes in the thickness of the ciliary body [1]. (Figure 1)

**Figure 2:**
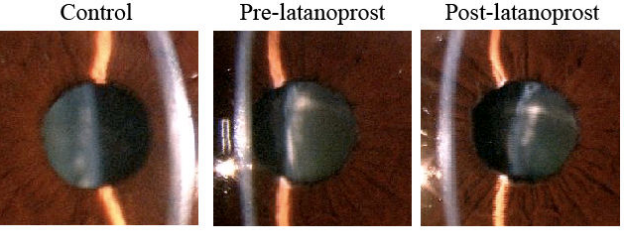
Change in lens position after topical latanoprost. Photographs before latanoprost (pre-latanoprost), and the movement of the subluxated lens posteriorly after latanoprost. Left panel shows a control eye.

**Figure 3:**
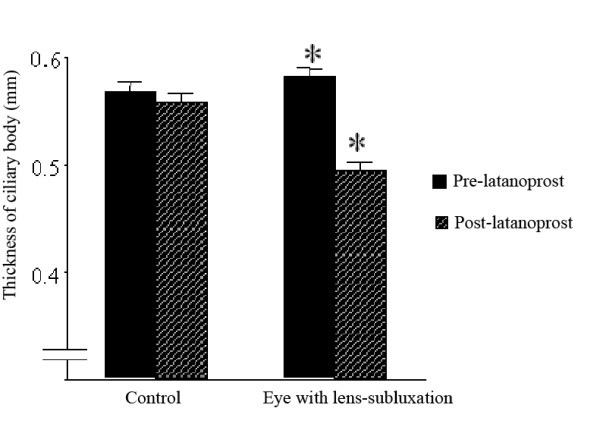
Relaxation of the ciliary body muscles after treatment of latanoprost. Before and after latanoprost on right eye, lens subluxation, thickness of ciliary body was measured in four directions, vertical and horizontal phase (average ± SD). Control means left eye, non-lens subluxation eye. (*: *P *< 0.01, paired *t *test).

One hour after one drop of 0.005% latanoprost, the right IOP decreased from 20 mmHg to 17 mmHg and the IOP in the left eye was reduced from 10 mmHg to 7 mmHg. Slit-lamp biomicroscopy showed a large empty space between the lens and iris indicating a movement of the lens posteriorly. The lens in the right eye had not shifted (Figure 2). In addition, UBM showed that the thickness of the ciliary body had decreased significantly (Figure 3).

Latanoprost is a prostagrandin F2-alpha receptor antagonist [2] that increases the efflux of aqueous humor through the uveoscleral route [3,4]. The increase results from a re-organization of the extracellular matrix including the matrix metalloproteinases (MMPs) [5]. In the pseudoexfoliation syndrome, changes in the MMPs are associated with the loss of the zonules of Zinn fibers. Latanoprost is widely used to reduce the intraocular pressure (IOP) in eyes with glaucoma, [6] and latanoprost has been used safely as a first line therapy in eyes with pseudoexfoliation glaucoma [7,8]. Our patient with the pseudoexfoliation syndrome and subluxated lens offered us an opportunity to examine the effect of topical latanoprost on the position of the lens.

The presence of desquamative material on the zonules of Zinn fibers can lead to abnormalities which may account for the subluxation. The increased aqueous humor efflux through the uveoscleral route by latanoprost is probably aided by the relaxation of the ciliary body muscle [9-11]. In our case, a decrease in the thickness of the ciliary body was detected by UBM. Although a previous report states that the mean ciliary body thickness increases two weeks after latanoprost administration [12], our data showed a rapid decrease in the thickness of the ciliary body in an eye with a subluxated lens. Approximately two-third of the anterior part of the ciliary body moved posteriorly which would increase the tension of the zonule of Zinn fibers [13]. Thus, latanoprost relaxes the ciliary body muscle and increases the tension on the zonule of Zinn as with topical atropine sulfate.

## Conclusion

We suggest that the subluxated lens was due to the loss of the zonule of Zinn fibers in the superior margin of the lens, and this loss would make it easier for the lens to move posteriorly. Although the movement of the lens was not sizeable, any increase in the distance between the cornea and lens will reduce the overall refractive power of the eye. UBM is useful to determine the mechanism of unexpected symptoms such as the monocular diplopia in our patient, and UBM should be considered for patients with pseudoexfoliation syndrome following topical medication. In spite of these changes, latanoprost can be used in patients with weakened zonules of Zinn, but careful follow-up examinations are recommended especially for lens subluxation.

## Competing interests

The author(s) declare that they have no competing interests.

## Authors' contributions

TK examined the patient and drafted the manuscript. MT examined the patient. YK performed a literature review. All authors read and approved the final manuscript.

## Consent

Written informed consent was obtained from the patient for publication.
